# Patients’ experiences of continued treatment with extended-release naltrexone: a Norwegian qualitative study

**DOI:** 10.1186/s13722-022-00317-2

**Published:** 2022-07-18

**Authors:** Anne Marciuch, Ida Halvorsen Brenna, Bente Weimand, Kristin Klemmetsby Solli, Lars Tanum, Bente K. Røstad, Bente Birkeland

**Affiliations:** 1 Department of Research and Development in Mental Health, Akershus University Hospital, PB. 1000, 1478 Loerenskog, HF Norway; 2 Department of Medicine, Faculty of Clinical Medicine, University of Oslo, Oslo, Norway; 3 Department of Addiction Medicine, Haukeland University Hospital, Bergen, Norway; 4 Department of Clinical Psychology, University of Bergen, Bergen, Norway; 5 Department of Health, Social and Welfare Studies, University of South-Eastern Norway, Drammen, Norway; 6 Norwegian Centre for Addiction Research, University of Oslo, Oslo, Norway; 7 Vestfold Hospital Trust, Toensberg, Norway; 8 Faculty for Health Science, Oslo Metropolitan University, Oslo, Norway; 9 RIO-a Norwegian users’ association in the field of alcohol and drugs, Oslo, Norway; 10 Department of Psychosocial Health, University of Agder, Kristiansand, Norway

**Keywords:** Extended-release naltrexone, Opioids, Opioid use disorder, Treatment of opioid dependence, Recovery, Qualitative, Patient experience

## Abstract

**Background:**

The opioid antagonist extended-release naltrexone (XR-NTX) in the treatment of opioid use disorder (OUD) is effective in terms of safety, abstinence from opioid use and retention in treatment. However, it is unclear how patients experience and adjust to losing the possibility of achieving an opioid effect. This qualitative study is the first to explore how people with opioid dependence experience XR-NTX treatment, focusing on the process of treatment over time.

**Methods:**

Using a purposive sampling strategy, semi-structured interviews were undertaken with 19 persons with opioid use disorder (15 men, four women, 22–55 years of age) participating in a clinical trial of XR-NTX in Norway. The interviewees had received at least three XR-NTX injections. Qualitative content analysis with an inductive approach was used.

**Findings:**

Participants described that XR-NTX treatment had many advantages. However they still faced multiple challenges, some of which they were not prepared for. Having to find a new foothold and adapt to no longer gaining an effect from opioids due to the antagonist medication was challenging. This was especially true for those struggling emotionally and transitioning into the harmful use of non-opioid substances. Additional support was considered crucial. Even so, the treatment led to an opportunity to participate in society and reclaim identity. Participants had strong goals for the future and described that XR-NTX enabled a more meaningful life. Expectations of a better life could however turn into broken hopes. Although participants were largely optimistic about the future, thinking about the end of treatment could cause apprehension.

**Conclusions:**

XR-NTX treatment offers freedom from opioids and can facilitate the recovery process for people with OUD. However, our findings also highlight several challenges associated with XR-NTX treatment, emphasizing the importance of monitoring emotional difficulties and increase of non-opioid substances during treatment. As opioid abstinence in itself does not necessarily equal recovery, our findings underscore the importance of seeing XR-NTX as part of a comprehensive, individualized treatment approach.

*Trial registration*: Clinicaltrials.gov # NCT03647774, first Registered: Aug 28, 2018.

## Background

The opioid antagonist extended-release naltrexone (XR-NTX) blocks the effects of opioids and is a promising, safe and effective [1], treatment for patients with opioid use disorder (OUD) [2–4]. By its long-acting effect it offers an opportunity for persistent abstinence from all opioids, including agonist medication prescribed through opioid treatment programs (OTPs). OUD is a chronic, relapsing disorder with serious consequences for the individual, their families and society, and illicit opioid use is a major global public health problem, taking an ever-increasing number of lives annually [5–8]. Despite promising results from clinical trials, many patients choose to discontinue XR-NTX treatment prematurely [9, 10], which may somewhat limit the clinical usefulness. Relatedly, the question regarding what patients need to be able to continue treatment and stay abstinent, in addition to being blocked in itself, remains unexplored. Such knowledge is crucial to tailor treatment and reach more people with OUD, and thus prevent the harmful effects of opioid addiction for the individual, family, and society. Many individuals with substance use disorders (SUD), hereunder OUD, use the substance as a means to escape reality and regulate emotions [11], underlining the importance of understanding how patients adjust to having the effects of opioids blocked.

Traditionally, recovery from OUD has been understood as abstinence [12], but likely also involves something beyond mere abstinence, such as improvements in health and wellness [13]. Achieving long-term abstinence from opioids can require both time and multiple efforts [14–16], highlighting the complex nature of opioid dependence and the importance of effective treatments. Medication for opioid use disorder (MOUD), with the opioid agonists buprenorphine or methadone, has long been the recommended treatment for opioid use disorders by the World Health Organization [17], and has been shown to reduce illicit opioid use, prevent relapse and reduce mortality [18–21]. In Norway the medical treatment of OUD is organized in an OTP system, overseeing treatment with opioid agonists, most commonly buprenorphine or methadone. Opioid antagonist treatment is currently not available except in clinical trials. Generally, and especially for young people, agonist medication is not the first choice of treatment for OUD in Norway, unless professionals judge it to be the most suitable and safe option [22]. In addition to MOUD, non-medication treatment for OUD is also available, e.g. in-patient detoxification or therapeutic treatment and out-patient counselling, despite studies showing that abstinence-oriented treatments have poorer outcomes than agonist treatment [23] when it comes to sustained abstinence and overdose risk after discharge [21, 24].

Despite being safe and effective [17], agonist medication, as well as the OTP system itself, is not without disadvantages. Use of illegal substances and alcohol alongside agonist treatment is not uncommon [25, 26] and poses risks to retention and long-term opioid abstinence [27]. Patients receiving agonist medication are frequently stigmatized [28], and control measures (e.g. daily pick up of medication, supervised intake or regular drug urine testing) are often mandatory [29], due to the need for control over the potential harmful societal effect of medication dispersion. In a Norwegian study [30], patients described the OTP system as overruling and degrading; a limbo between recovery and continued addiction. Also, not all patients manage to stay in, or even want agonist MOUD, e.g. due to non-conformity to the demands of the system, inability “play by the rules” [31], or side-effects of the medication, such as constipation, headaches or sedation [32]. For some, lasting abstinence [33, 34] and abstinence from *all* opioids, including opioid agonist medication, is the treatment goal [35].

XR-NTX treatment involves “being blocked” from the reinforcing, physiological and subjective effects of opioids over time [36, 37], which can reduce opioid use and sustain abstinence [9]. At the same time, being blocked also means giving up the desired (e.g. euphoric or sedative) effects of opioids. Still, XR-NTX is an option for people with OUD seeking abstinence from both illicit and prescribed opioids within the safety of treatment. The effects of opioids are blocked for a considerable, fixed length of time (approximately 4 weeks), which for many patients likely is important in contributing to a distance from opioids both psychologically, physically and temporally.

Several studies with XR-NTX have shown positive results. A previous randomized controlled trial [2] demonstrated that XR-NTX is as safe and effective as buprenorphine-naloxone (an opioid agonist). The results were consistent with other studies [4, 38–41], showing a decrease in opioid and substance use, improvements on psychosocial variables, and less opioid craving [3, 42–44]. The need for XR-NTX to be reinforced by psychosocial interventions or psychological treatment [45–47] has been emphasized, while a lack of psycho-social follow-up has been linked to treatment discontinuation [44].

Despite these promising results, few qualitative studies examining patients’ perspectives on XR-NTX exist. In one study [48] of patients’ perceptions of medications for OUD following release from jail, XR-NTX was perceived as a helpful, relapse-preventing intervention, albeit with limitations. A study examining patients’ perspectives on initiating treatment with XR-NTX [49], found detoxification, ambivalence, and fears regarding antagonist treatment to be barriers to treatment initiation. A recent study of induction to treatment among patients living with HIV and OUD [50] emphasized the importance of addressing patients’ expectations regarding induction to improve initiation rates. This is in line with a recent qualitative study focusing on patients discontinuing treatment [51]. The study indicated unfulfilled expectations as central to discontinuation, but also emphasized that the motivation for abstinence from illicit opioids remained after discontinuation.

No study, to our knowledge, has focused on patients’ experience of staying in treatment with XR-NTX well past the initiation phase. The aim of this study was to explore and describe how people with opioid use disorder experience treatment with XR-NTX over time, including the possible benefits, challenges, and needs that arise during treatment. This study offers a unique opportunity to further the understanding of what makes patients continue treatment or what obstacles need to be overcome, and thus, how to facilitate treatment initiation and course, and increase utilization.

## Methods

This study employed an explorative and descriptive qualitative design. Analysis was not pre-registered, and the results should be considered exploratory. A semi-structured interview-guide with open-ended questions was developed (AM, BW) with input from co-researchers from “RIO-en landsdekkende brukerorganisasjon på rusfeltet” (“RIO-a Norwegian users’ association in the field of alcohol and drugs”), and proLAR Nett (“Pro-OTP Network”), a national organization of people in OTPs. Interviews were analyzed using an inductive content analysis inspired by Elo and Kyngas [52], and Graneheim and Lundmann [53].

This qualitative study is a sub-study of the Norwegian, naturalistic, multicenter, clinical treatment study “Long acting naltrexone for opioid addiction: the importance of mental, physical and societal factors for sustained abstinence and recovery” (NaltRec). The overall NaltRec study included 162 persons, 39 female, and 123 male, aged 18–65 years, with a diagnosis of OUD. All participants were voluntarily seeking treatment with XR-NTX, and were recruited through OTP counselors or other health care workers either at addiction clinics or in the community health services, by study personnel at the detoxification units, or through newspaper articles.

After inclusion to the trial, participants went through complete detoxification from opioids, before receiving an injection of XR-NTX which they subsequently received every 4 weeks during the 24 + 28 week study period, together with multiple assessments. The NaltRec study was conducted at five urban (population > 40.000) addiction clinics throughout the southern part of Norway. The fifth site joined the study at a later date than the first four, and was not present when the qualitative sub-study was carried out. The catchment areas included close to half of the total population in Norway. Treatment with XR-NTX was not generally available in Norway when the study was conducted. For further details on the NaltRec study, see Weimand et al. [1].

The qualitative sub-study of NaltRec consisted of interviews with 32 participants; 13 who had received at least one injection, but chose to discontinue treatment before 12 weeks, and 19 who chose to continue treatment for at least 12 weeks (receiving at least 3 injections), constituting the sample for the present study. Both samples were interviewed using the same interview guide. 102 (63%) of the original sample of 162 in the NaltRec study chose to receive at least 3 injections. Study personnel mediated contact with participants in NaltRec who had given written consent to be individually interviewed, and met the inclusion criteria of being in continuous, active XR-NTX treatment for at least 12 weeks (three injections) after inclusion at the time of interview. Participants were informed that not all consenting to be interviewed would actually be contacted. When recruiting we used a purposive sampling strategy. The selection of participants was strategic, based on inclusion criteria, as well as aimed to include a balance in gender, and geographic spread in the four sites participating when recruiting. All patients contacted initially agreed to be interviewed. However there were 10 patients who either withdrew consent, or who we were unable to reach subsequently. We had initially aimed to interview 20 patients, as 20 participants were considered feasible to recruit within available time and resources, and also sufficient with regard to the topic and scope of the study, as well as the planned length of each in-depth interview which allowed for ample information from each participant.

We conducted interviews with 19 participants—15 male and four female. Although our sample had a gender imbalance, due to difficulties recruiting females during the inclusion period, it reflects the gender imbalance present in the overall study (24% female), as well as the gender imbalance in OTPs in Norway [54] and among treatment seeking individuals with OUD in Europe [7], as well as the historically higher SUD prevalence in men [55, 56]. The mean age was 38 years (range = 22–55 years). Thirteen participants were in OTPs prior to NaltRec study participation. Participants had received between three and 12 injections at the time of the interview (Table 1).

**Table 1:** Participant characteristics

Characteristic	Total N = 19
Age, mean (range), years	37.95 (22–55)
Sex, n (%)	
Male	15 (78.9)
Female	4 (21.1)
Ethnicity, n (%)	
Norwegian	17 (89.5)
Other	2 (10.5)
In OTPs previous to study participation, n (%)	13 (68.4)
Most common living arrangements last three years, n (%)	
Alone	12 (63.2)
With partner	4 (21)
With parents or other family	3 (15.8)
Currently living with someone with problematic drug/alcohol use**,** n (%)	0 (0)
Years of completed education, mean (SD)	13.4 (2.5)
Age at start of regular opioid use (yr), mean (SD)	25.4 (9.0)
Length of regular opioid use (yr), mean (SD)	11.9 (7.0)
XR-NTX injections received, mean (range)	7 [3–12]
Number of XR-NTX injections received at interview time, n	
3	1
4–6	6
7–9	8
10–12	4

The interviews focused on different themes relating to the participants’ experience of receiving XR-NTX; “Motivation for XR-NTX-treatment”, “Experience of being blocked”, “Barriers and enablers in XR-NTX-treatment”, “Mental and physical health”, “Care and support”, and “Quality of life and recovery”. Each topic consisted of three to six “core questions” supported by prompts to allow for details and elaboration. To keep the exploration close to participants’ everyday life, we asked for examples. Interviews were conducted face to face in a private room by the core research group (AM, IHB, BB, BW) and trained personnel, and lasted 60–90 min. All interviews took place between April 2019 and February 2020. Only one interview was conducted with each participant on account of choices made in the overall study, as well as due to restrictions in time and resources. However, we also expected this design would give us the opportunity to go in-depth into each informant’s reflections regarding both past and present experiences with XR-NTX. In addition, a variability in length of treatment between individuals was sought, ensuring longitudinal examination of the experiences of treatment.

Interviews were audio-recorded and transcribed verbatim (AM, IHB, study personnel). Transcripts were imported into NVivo 12 [57] for systematic coding. To ensure credibility and the trustworthiness of findings, two of the core research team members (AM, BW) analyzed the findings. Theoretical saturation [58] was not considered as the material was analyzed after the data collection phase was finished.

An inductive approach [52, 53] was used for coding, keeping codes close to the text. Subsequently, codes were grouped into categories, based on the common denominators that were identified. Codes were reorganized several times to achieve “a full description” of the participants’ experiences, and consensus between three of the authors (AM, BW, BB). Categories were thoroughly discussed, and after scrutinizing and re-organizing, we reached agreement in grouping the categories under three themes. In reporting the findings, quotations have been selected to illustrate findings and to ensure trustworthiness.

Ethical approval for the NaltRec trial, including the present study, was granted by the Norwegian Regional Ethical Committees for Medical and Health Research Ethics (REK) committee South East A (# 2018/132), by the personal data protection representative for each of the sites, and by the Norwegian Medicine Agency (NOMA), EudraCT Code 2017-004,706-18. The trial is registered on Clinicaltrials.gov # NCT03647774. It was first registered on Aug 28, 2018, before first participant inclusion on Sep 21, 2018 [1]. All participants gave written, informed consent for their participation.

Interviews were conducted by personnel not involved in the follow-up of the interviewed participant in NaltRec. No information from interviews was shared with clinical or study staff. Interviews were transcribed verbatim, anonymized, and stored at a secure server at the sponsor hospital. Participants were given fictitious names in both the transcripts and in the quotes included in this article.

## Findings

**Fig. 1:**
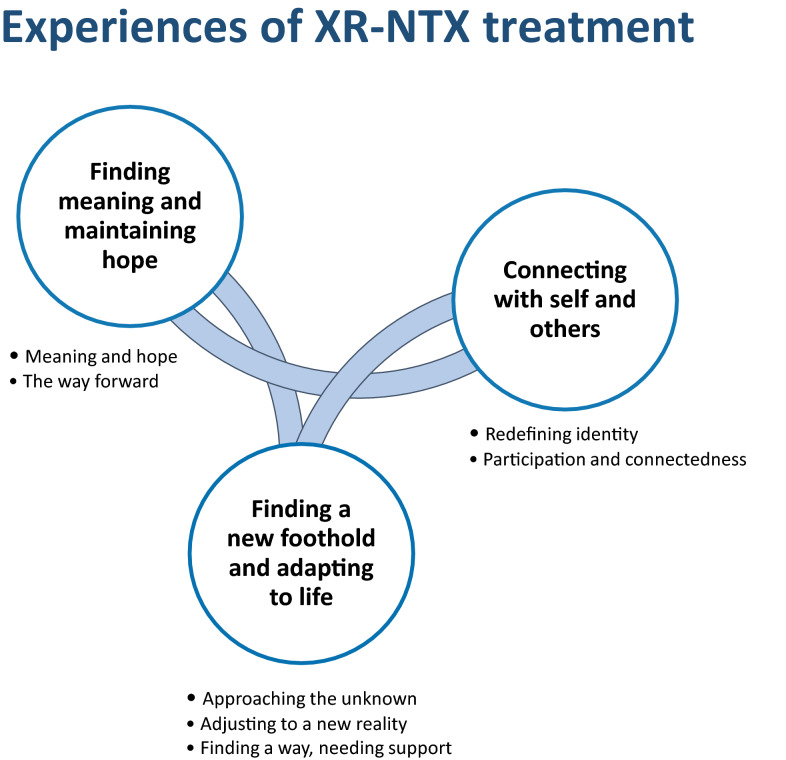
Categories and subcategories

When exploring participants’ experiences with XR-NTX, three themes were formulated: “Finding a new foothold and adapting to life”; “Connecting with self and others”; and “Finding meaning and maintaining hope.” (Fig. 1). These were derived from organizing and grouping the sub themes together into themes describing important aspects of the treatment process, and are considered to be a valid description of all participants’ experience. The themes describe the process and experiences of treatment with XR-NTX, but are not necessarily chronological, as they may assert themselves repeatedly and at different points during the treatment process. Except for the subtheme of “Approaching the unknown”, which is clearly connected to the initiation of treatment, the other themes and sub themes, e.g. the “adaptation” to a new life, or the maintaining of hope, represent aspects or issues that take place throughout treatment, and not only in either the initiation, maintenance or post-treatment phase. When participants discussed their experiences with XR-NTX it became clear that the process is not straight-forward, and that different benefits, challenges and needs might vary throughout treatment.

## Finding a new foothold and adapting to life

Even though XR-NTX was chosen voluntarily, being in treatment could be tough. Participants described having to find alternative ways to deal with challenges, which some found difficult, whereas others appreciated not being able to use opioids as a coping strategy and having to “…*resolve things like everyone else, (…) instead of escaping*” (Daniel, age 24, 9 injections).

### Approaching the unknown

XR-NTX is not an established treatment in Norway, and participants starting XR-NTX described entering a somewhat unfamiliar landscape. Nonetheless, the motivator of being free of opioids and/or the OTP system was strong, and the possibility of not experiencing opioid cravings was welcome.

Almost all participants experienced adverse effects around the detoxification and induction on XR-NTX, albeit to varying degrees in both duration and intensity. For some, symptoms such as pain or sleep disturbances persisted, but overall, participants described improvements in physical health after the initial phase.

The start-up was emotionally hard, but also positive because it involved an appreciation of a newfound emotional clearness. Moreover, being able to react “normally,” such as feeling empathy, happiness, and even sadness, was appreciated. Some of the participants, however, struggled with overwhelming emotions and found their new situation to be unmanageable. The strength of these emotions could be shocking; one participant reported that “*All the emotions suddenly hit me like a train.*” (Tina, age 26, 4 injections).

After these initial challenges, quitting opioids involved positive changes, e.g., in physical- and cognitive functioning or a general sense of improved mental health, including feeling stable and more in touch with one’s emotions. Yet, although most participants experienced improved psychological functioning, some described struggles to deal with mental challenges or underlying issues that reappeared. One participant explained that he had experienced traumatic events and reported having *“so many thoughts in my head I’m unable to suppress, without [opioids]…”* (Roger, age 36, 6 injections).

Adjusting to a new reality.

“*Even though you start naltrexone you are not finished, the road continues.*” (Jon, age 44, 5 injections).

The commencement of XR-NTX treatment did not indicate a problem-free life. Adjusting to an existence without opioids could be encouraging, but no longer having the safety of the OTP system could be challenging. On the one hand, “being blocked” could be wholly unproblematic and mean diminished craving; as opioids “would not work anyway,” participants felt they no longer had to use any energy thinking about them. On the other hand, XR-NTX could feel like a prison. For some participants, opioids—especially heroin—had been an escape from reality, and XR-NTX represented losing this.

*“Seventy to eighty percent [of the time] I have felt safe (…), that no matter how things are in the future, you can’t use heroin, you’re blocked, you just have to deal with it (…). At other times (…), I feel imprisoned, like what the hell am I supposed to do*?!” (Gunhild, age 35, 6 injections)

Some participants reported trying heroin, mainly to see if XR-NTX really blocked its action and experienced that it did. Several described hearing of ways to break the blockade, and although one participant described succeeding in this, most did not welcome this information as it could induce cravings.

Participants described generally using fewer non-opioid substances after inclusion in the NaltRec study. Some participants reported using no substances, and some described a limited non-opioid use that was typically less problematic than their previous opioid use had been. A few participants however, found being without opioids to be unmanageable; feeling desperate for the opioid effect, resulting in serious and harmful non-opioid use. As Gunhild explained, she had previously identified as a “*heroin* addict”, not a “*drug* addict”, and the use of other (unfamiliar) drugs was a complete surprise.

“*It hadn’t crossed my mind that I would do it. And maybe that was naïve of me. Because I know I like to escape from myself. Yeah, I thought maybe I would get drunk from time to time or something like that.*”

However, suddenly again “struggling with myself”, Gunhild, who was well acquainted with fluctuating, long-term psychological difficulties, found it unbearable to manage without anything (i.e. opioids) dulling the emotional pain, thus lapsing into a serious and harmful use of non-opioids.

### Finding a way, needing support

Finding a way to manage an opioid-free life was significant. Participants voiced the need for something more than “just the injections”, such as support or some activity.

“*If I didn’t have a job, and got naltrexone, then it wouldn’t matter, if I just sat at home and watched TV. Because half of what I did when I didn’t have a job, to make time go by, was to use drugs.*” (Daniel, age 24, 9 injections)

Still, not expecting too much too fast was emphasized; just getting used to being sober could be enough.

“*Most think, “I’m sober now, [I must get a job].” Then you’re starting too high up on the ladder, right, and then you’re bound to fall.*” (Thomas, age 42, 9 injections)

Although some clearly wanted to manage on their own, participants generally saw the value of some kind of support from external services, which many already had. The first months, in particular, were underlined as a period where extra support was needed.

“*I needed [help], to understand how I was supposed to cope without looking for something* *that could dull everything. (…) There should be more follow-up, especially in the first two months. I really think so, because I felt so very alone.*” (Karl, age 54, 8 injections)

The discontinuation of opioids could also highlight the need for additional help, and the handling of underlying mental issues was emphasized as important, as these could stand in the way of fully utilizing the potential of the XR-NTX-treatment and of further recovery. In addition to various services, some also voiced a wish for peer support groups with other patients receiving XR-NTX. Several participants found the monthly visits of the NaltRec study useful as an opportunity to reflect on the previous month.

The feeling of support from friends and family—having someone to reach out to if things got difficult—was important. However, having made the decision to quit opioids also involved expectations of continuing to “do good,” which was described as a possible barrier to actually utilizing the potential support system.

“*Mum doesn’t know I use amphetamine now (…). It’s hell not being able to tell mum I’m struggling.*” (Roger, age 36, 6 injections)

Participants generally did not want to involve family in the treatment, not wanting to overtax relatives or feeling that it was nobody’s business but their own. However, for some, support from family had “always” been in place, and choosing to start naltrexone was thus a way of “giving back;” showing that a step forward had been taken.

Some participants described that what health services could offer was not what was needed, and proposed the value of peer-support from other patients who had experience with XR-NTX.

“[*I wanted to] establish a group for those of us using naltrexone, (…) so we can meet and have a coffee and discuss things. NA [narcotics anonymous] says something about it; share strength, hope and experience. Because if you don’t, you are horribly alone with your problems*.” (Harald, age 50, 7 injections)

## Connecting with self and others

*“[Heroin] takes a piece of your soul, a piece of your identity.”* (Roger, age 36, 6 injections).

An important aspect of XR-NTX treatment was that it offered the opportunity to be “normal”; leaving stigma behind, participating in society, and reclaiming the self.

### Redefining identity

Quitting opioids allowed for the rediscovery of oneself, which could be both liberating and unpredictable.

“*What has surprised me most after starting up is that I have found myself again, got to know myself, but at the same time it’s been quite an insecure process.*” (Jon, age 44, 5 injections)

The need for opioids had previously overshadowed everything, and as Jon explained, having used opioids for a long time could mean uncertainty about one’s present identity: “*Who am I today*?” Some expressed feelings of having changed, in preferences or interests, or lost parts of themselves they previously valued. Still, most felt they were moving towards who they wanted to be. No longer being dependent of opioids, and belonging to “the normal group” contributed to a sense of dignity as it also meant escaping the stigma of being an “OTP patient” or “drug addict”, a distrusted outsider. Feeling useful and being able to take responsibility meant growing as a person. The accomplishment of quitting opioids or the OTP further contributed to a sense of pride and increased self-esteem.

### Participation and connectedness

Belonging and being able to participate in society was considered important. However, functioning in the world with little or no experience of operating in a “normal society” was also challenging. Still, several participants described suddenly feeling like part of something when they had a job, had more time and energy to participate, or experienced strengthened relationships; “*I’ve always had my family. Before, I was just there, now I am* with *them*.” (Anders, age 34, 10 injections).

An increase in social needs could be challenging, as it could involve going out more, and disregarding the previous structure in their life. Nevertheless it could also be beneficial, signifying freedom from an almost compulsive routine and the freedom to participate in social events, and society.

“*Before I couldn’t… Or, I probably could have if I planned it, but then it had to be part of that plan.”* (Kevin, age 26, 7 injections)

### Finding meaning and maintaining hope

“*It has changed my life; it’s a whole new world.”* (Kevin, age 26, 7 injections).

Although the exact reasons for starting treatment and future goals varied, hope for a better life was shared. Dissatisfaction with the regulations of the OTP or the medication itself was common and combined with the desire for an opioid-free life was a strong motivator for attempting XR-NTX.

### Meaning and hope

Many participants described having felt there was little hope, and not feeling free as long as they were using opioids. Being on XR-NTX offered a new perspective; it allowed for a future for the first time in a while. XR-NTX could also be the endpoint in a long process, providing the means to finalize the hope and ambition of a “normal life.”

The positive changes taking place during treatment seemed to fuel hope and contribute to optimism about the future. Although various difficulties could make hope waiver, it was not lost, and the decision to continue treatment seemed strong. Many participants felt they led a more meaningful life.

*“Yes, there is meaning to life. There are many things I want to do now, that I had stopped more or less, like fishing, being outdoors, and getting in shape. Everything feels more meaningful.”* (Tor, age 43, 7 injections)

However, maintaining hope could prove difficult if things did not turn out as imagined. Having previously seen XR-NTX as a miracle, expectations turned into broken hopes when reality turned out differently.

“*Maybe I should just give up and accept that I’m an addict, and that’s what I’ll be for the rest of my life.*” (Tina, age 26, 4 injections)

### The way forward

The time limitation of XR-NTX treatment felt frightening for some, as the trial was limited to one year, and there was no possibility of continuing treatment after the end of the trial. Although achievements had been made, for some, thinking about “being back on my own” created uncertainty about the risk of relapsing to opioids and further destroying what had been achieved. Others felt that the distance from opioids, attained during treatment, was vital. Although they initially had feared relapse and the end of the treatment, having dealt with life without opioids for nearly a year, they felt these were no longer of concern. Yet, even though much had been accomplished, there was sometimes still a sense of uncertainty about the future, with participants not being sure if the progress would continue once nothing was “holding them back”.

“*It remains to be seen when I’m actually done, after the 13 injections. That’s another task to overcome, right?*” (Marius, age 26, 4 injections)

There were also some worries about not being able to achieve abstinence from all substances during treatment, which gave rise to distressing thoughts:

“*What will happen if I’m still caught up in drugs and relapse to heroin? What will happen to me then? Is it back to the OTP, and Subutex, and all that? I don’t want that. (...) If that happens, I might as well shoot myself, because I don’t want to go back.*” (Roger, age 36, 6 injections)

For patients struggling with a harmful use of substances, the question of whether a return to heroin would be less harmful than the use of various other substances was raised. However, beyond this, participants in the present study did not voice any doubt as to whether or not to continue XR-NTX during the trial.

## Discussion

To our knowledge, this is the first study exploring in-depth the experiences of people with opioid dependence successfully inducted and choosing to stay in treatment with XR-NTX over time. This qualitative study offers five major findings. First, the induction to treatment held considerable challenges for patients. This was mainly related to the difficulty of handling the emerging emotions when discontinuing opioids. Second, XR-NTX was experienced as an effective treatment, signifying freedom from both illegal opioids and from the “OPT system”, and involving substantial benefits for patients, such as improved health, or the opportunity to participate in society. Third, for a few patients, extensive use of non-opioid illegal substances could indicate considerable challenges dealing with opioid blockade and be a substantial barrier to continued XR-NTX treatment and further recovery. Fourth, mental health problems could also be a considerable barrier to the treatment process, patients not being able to use opioids for symptom relief. Fifth, our results underscore the need for individualized and tailored support and follow-up during XR-NTX treatment.

Our findings are in line with qualitative studies showing that patients consider XR-NTX a relapse-preventing option reducing cravings [48, 49], as well as previous studies concluding that XR-NTX is an effective and feasible treatment for OUD [2–4]. Whether patients struggled greatly with the opioid antagonism, or experienced it as unproblematic, XR-NTX was still overall perceived as life-changing, and the desire to continue was strong. This was mainly connected to escaping what was described as detrimental aspects of the OTP system, as well as leaving dependence behind and no longer having the compulsion to take opioids at the cost of other pleasures or interests. However, the findings also highlight that XR-NTX treatment did not have the desired outcomes for all, and in many cases required additional professional, social and/or familial support. The study answers some of the questions regarding the psychological aspects of opioid receptor blockade, and sheds light on possible hindrances and facilitators for staying in treatment over time. These factors have been identified as important areas for investigation by patients and organizations for people with SUD [1].

Interestingly, few, if any, *external* barriers to treatment retention and heroin abstinence were mentioned by participants, such as economic insecurity or social factors. This is contrary to previous findings [48], and may at least in part be attributable to the Norwegian welfare society, securing inhabitants a minimum standard of living, including free health care and social services. Participants were recruited from a naturalistic outpatient treatment setting, and were highly motivated for XR-NTX. As mentioned, the majority of our participants were previously in OTPs, which is widely accessible at no cost in Norway [7]. Thus the interest to start XR-NTX likely signifies a specific motivation for the antagonist effect of XR-NTX and further recovery, rather than economical motivations or desperation due to having no other alternatives.

### Tailored support

In line with a biopsychosocial model of addiction [59], our findings illustrate that OUD is a complex, multifaceted phenomenon, necessitating a diverse and individualized treatment approach. People struggling with OUD need concurrent focus on a wide range of domains, e.g. in medical, psychological, emotional, relational, motivational, occupational, and social issues. Both our findings and those of previous studies [44, 46, 47, 60] emphasize the need for something “in addition” to the monthly blockade of opioid receptors.

One size does however not fit all, illustrated by the variability in participants’ experiences and diverse needs for support during treatment. While some participants were undeniably in need of increased enforcement, others clearly desired the autonomy XR-NTX offered, preferring no service involvement. Regardless, the induction and early weeks of treatment will probably be a period where enforced support will be beneficial for most patients. It is also important to note that for some patients, measures beyond basic support will initially be excessive, as merely adjusting to an opioid-free everyday life poses sufficient challenges to overcome.

For many participants clearing up and experiencing the outside world and inside emotional states more intensely felt liberating, yet this freedom could also come at a cost, in the form of increased emotional vulnerability and an increased need for additional support. The various areas requiring strengthened efforts ranged widely, which needs to be taken into consideration when planning XR-NTX treatment course. While some participants expressed a need for health services, such as mental health treatment or addiction and motivational counselling, others just wanted to have something to do, a job, someone to chat with, or to give or receive support from peers with experience of XR-NTX treatment. Interestingly, patients were hesitant to involve family or friends in the treatment, and although their support was valued, the importance of reinforcement from health- or social services was emphasized. Not surprisingly, our results emphasize that additional measures are more important than ever. Although XR-NTX addresses the physiological basis of addiction, the sole blocking of opioids is insufficient alone.

### Use of opioids and other illicit substances

Consistent with other studies of XR-NTX, participants overall reported a reduced use of illicit opioids [3, 4, 40]. Still, also in line with other studies [48, 61, 62], some participants described instances of opioid use, mainly to “test the blockade.” Contrary to previous findings [62], repeated challenges to the blockade did not seem to be a warning sign for risk of relapse to polydrug use and crime. Rather, testing the blockade seemed to have minor impacts on functioning, and did not drive thoughts of discontinuation, which is in line with previous qualitative findings [48].

Our participants also described an overall reduction in non-opioid substance use, however, a few reported an increase. Many patients with OUD have polysubstance-use disorders [63, 64]. There are indications that frequent substance use is common in OTPs [65, 66], and that agonist treatment, although associated with lowered opioid use, to a lesser degree is associated with lowered polydrug use [25]. However, studies have also shown that substance use, both opioid and non-opioid, appears to decrease over time in agonist treatment [67]. The picture is likely similar for XR-NTX; when comparing XR-NTX to buprenorphine in a clinical trial over 12 weeks, no overall change in the use of most substances was found [2]. This qualitative study provides some elaboration and additional nuances on non-opioid use during XR-NTX treatment, capturing details in individual experiences.

Some patients, when unable to handle life without opioids and desperate for an escape, may turn to “anything” (i.e. other substances) if there is no prospect of an opioid effect. For our participants, an extensive use of non-opioid substances carried the likelihood of harmful effects on health and functioning and could raise the question whether continued XR-NTX treatment would be sustainable over time if unable to get this use under control. Even though XR-NTX meant avoiding the detrimental effects of opioid dependence, the effects of increased non-opioid use carried disadvantages which could be as great as, or even greater than when previously using opioids. In addition, further recovery and the building of a new life seemed difficult with such a use. Interestingly, participants still preferred to continue XR-NTX treatment, which likely points to the strength of the hopes tied to XR-NTX treatment.

A pronounced desperation for an escape and the turning to other substances will likely be a warning sign that for the patient opioid antagonism is very challenging to handle, and that additional measures are needed to continue to benefit from continued treatment. Non-opioid use is an important outcome measure of XR-NTX treatment, and clinicians should remember that XR-NTX treatment does not automatically mean overall abstinence. An extensive use of non-opioids will in many cases complicate further recovery and raises the question of how to achieve “freedom from opioids” without the cost of introducing or increasing use of harmful substitute drugs.

### Experience of opioid blockade

For many, the struggle of “not being able to escape into opioids,” an unnerving prospect initially, was not an issue after having passed the early phases of treatment. This might in part be a form of defense mechanism, making it psychologically easier to write off and accept not using opioids when there is no conceivable possibility of intoxication by opioids, which in turn in itself might diminish craving. In addition, the majority of our participants, having previously been in OTPs had probably not used the opioid agonist medication as a means of escape. Thus XR-NTX, instead of feeling like an obstacle to escape, felt mainly as a safety net, guarding against relapse. Others, however, struggled visibly. This may to some extent be understood from a developmental-learning perspective, which also underlines the importance of support when learning and implementing new habits [68]. “Quitting drugs” involves not only breaking old habits, but also learning new, such as the regulation of emotions without the use of opioids. This might prove difficult for some, whereas for others, the ensured abstinence from opioids over time might enforce the learning of new habits and further abstinence.

Based on our findings, mental health difficulties are of importance for how well opioid blockade is handled, coinciding with findings that addressing mental problems in patients with OUD likely enhances the odds of a stable recovery [15]. Comorbid psychiatric disorders are common among people with SUD [69, 70], and contribute negatively to the course and treatment of OUD [71]. Furthermore, previous physical or sexual abuse and comorbid mental disorders are associated with the persistence of opioid use [16], and higher scores of anxiety and depression seem to be concurrent with increased difficulty reducing illicit substance use [42, 72]. However, this association needs further investigation, as not all participants with mental health difficulties found being blocked difficult. In addition, symptoms of anxiety, depression, and insomnia have been shown to be improved by XR-NTX-treatment [42]. Still, for some, lack of adequate psychological treatment and support systems, both before and after starting XR-NTX treatment, might complicate the course of treatment.

How being blocked was experienced did however not seem to be wholly explained by factors like the amount of support offered or received, or the individual’s mental health, and was not constant, but could vary within the same person, from “ok” to “like a prison.” Likely, differences in social or psychological factors, like personality traits or coping skills are at play, as well as varying life conditions or circumstances. This again highlights the complexity of opioid dependence and the challenges people with OUD face, underscoring the need for diverse approaches and varied professional expertise to help meet these challenges. Still, a more systematic exploration of both external and internal factors associated with how being blocked is experienced and tolerated, and the connection with e.g. treatment outcome and success is needed to conclude further.

### Treatment length

XR-NTX-treatment is not intended to be lifelong and there is no recommended standard treatment-length [73]. The limitation of treatment to one year in this study might imply a positive expectation that the person with OUD will be able to manage independently after this period, strengthening agency and self-belief. On the other hand, some patients might need extended time in treatment [44], and our findings indicate that treatment length should be tailored to individual needs, rather than restricted to predetermined time periods. The commencement of treatment that is time-restricted might prove difficult for those who experience unsatisfying progress during the treatment course.

For many, opioid abstinence sustained by XR-NTX enabled a new, more meaningful life. This might be particularly important as SUDs impair many aspects of a person’s life, and might especially restrict the ability to experience meaning in life. Finding meaning is important to recovery in general [74], and has been associated with longer periods of abstinence from substance use [75]. Developing a meaningful life and finding a purpose in life is an important part of recovery from addiction [75–77]. Also, despite the serious, detrimental effects of non-opioid substance use, or the struggle to deal with emotional issues, the positive, life-changing effects of XR-NTX as well as the desire to continue treatment was strong. This was connected to the blocking of opioid effect and the resulting indifference to using them, constituting at least one less problem to handle, whether one had few or many other problems.

As XR-NTX offers a unique opportunity to avoid the effects of opioids over time, we suggest that it is what is achieved during treatment in terms of rehabilitation and recovery, rather than treatment duration or opioid abstinence in itself that is significant. Specifically, it seemed that the positive changes taking place during treatment were of more importance for participants than the abstinence from opioids per se, even though abstinence enabled these advances. This is in line with findings [78] showing that people with OUD are motivated by improvements beyond abstinence, such as better relationships, health, and meaningful everyday lives. These goals seemed, at least in part, to have been met for our participants through XR-NTX treatment.

## Strengths and limitations

This study’s explorative design offers important in-depth insights into XR-NTX as a treatment option for OUD. Participants were recruited from an open-label naturalistic treatment study, ensuring real-world relevance. Although this qualitative study included a limited sample, the material was rich in content, and presents detailed variations in experience. Both participants highly satisfied, as well as those disappointed with XR-NTX treatment participated, and gave voice to the nuances of the experience of treatment. Although our sample has a gender imbalance, this imbalance is in line with that of the overall study and that of treatment seeking persons with OUD in Europe, and we did not seek to address or explore gender differences explicitly.

Participants were interviewed at different times during the 12-month treatment period, which might influence what was emphasized or recalled. However, the themes presented were independent of the point in time at which the participants were interviewed. Still, the present study does not examine whether participants chose to stay in treatment beyond the time of the interview. Thus we do not know if some of the challenges mentioned were overcome, or if they better represent the narratives of patients choosing to discontinue treatment [51]. In addition, participants were interviewed only once. Multiple interviews with the same people throughout the course of treatment would perhaps have allowed for a better longitudinal examination, as well as captured treatment trajectories and the possible discontinuation among participants. At the same time, the single interviews allowed for an in-depth exploration of several individuals, at different times during the treatment period, and gave insight into the experiences of patients currently in treatment, in line with the study aim.

Participants might have been particularly motivated for the new treatment approach that XR-NTX represents in Norway, or have had a particular dissatisfaction with agonist treatment. Caution should be taken regarding the generalizability of our findings; nevertheless, they are transferable to similar settings, as a detailed description of the study’s context has been given, and patients freely choosing XR-NTX will likely share the same motivation. A detailed description of the research process has been given, ensuring transparency. It is also worth emphasizing that retaining patients in XR-NTX treatment over time can involve considerable challenges [9]. The patients in our sample had stayed in treatment for at least 12 weeks, many of them considerably longer. Thus this study offers a unique and interesting perspective on XR-NTX treatment over a longer period of time, and offers insights into the benefits and challenges of treatment over time.

## Conclusions

XR-NTX treatment, although potentially life-changing, can also involve serious challenges for patients. This means individuals receiving XR-NTX treatment will need additional services and support, especially in the beginning of treatment, but also when it comes to difficulties handling emerging thoughts, feelings and experiences when blocked from the effects of opioids. A strengthening of health- and social services, and emotional support whether from such services or from the patient’s network seems essential. Further, the monitoring of and assistance with arising emotional difficulties, as well as the possible subsequent harmful use of non-opioids is vital to help patients cope, and stay in treatment over time. Nevertheless, patients need for help will vary between people, at different times and with varying circumstances, which highlights the importance of seeing XR-NTX as part of a comprehensive, individualized treatment approach.

## Data Availability

The datasets generated and analyzed during the current study are not publicly available due to the possibility of compromising individual privacy and anonymity, but are available from the corresponding author on reasonable request.

## Abbreviations

MOUD: Medication for opioid use disorder
NOMA: Norwegian medicine agency
OPTs: Opioid (agonist) treatment programs
OUD: Opioid use disorders
SUD: Substance use disorders
XR-NTX: Extended-release naltrexone
